# Reinfection rate of hepatitis C in HIV-1 positive men who have sex with men: A systematic review and meta-analysis

**DOI:** 10.3389/fpubh.2022.855989

**Published:** 2022-07-29

**Authors:** Zhengwei Wan, Ping Sun, Emmanuel Enoch Dzakah, Liping Huang, Ping Shuai, Yuping Liu

**Affiliations:** ^1^Department of Health Management Center and Institute of Health Management, Sichuan Provincial People's Hospital, University of Electronic Science and Technology of China, Chengdu, China; ^2^Department of Molecular Biology and Biotechnology, School of Biological Sciences, College of Agriculture and Natural Sciences, University of Cape Coast, Cape Coast, Ghana; ^3^Guangdong General Hospital, Guangdong Academy of Medical Sciences, Guangzhou, China

**Keywords:** HCV, HIV-1, MSM, reinfection, rate

## Abstract

**Purpose:**

A reduction of 80% in new Hepatitis C virus (HCV) infection is expected by 2030. However, high HCV reinfection rates have been reported among the high-risk population. This meta-analysis aimed to assess the HCV reinfection rate after successful treatment of HIV-1 coinfected MSM populations.

**Methods:**

Bibliographic databases were searched and a random-effect model was utilized to calculate the pooled HCV reinfection rate. Sub-group and meta-regression were used to explore heterogeneity among selected studies. A funnel plot and Egger's regression test were performed to estimate the publication bias.

**Results:**

Sixteen studies with 9,017.2 person-years (PY) follow-up were included. The overall HCV reinfection rate following successful treatment among HIV-1-infected MSM was 5.27/100 PY (95% CI, 3.98, 6.96). Lower reinfection rates were observed in developed parts of Europe (5.28/100 PY; 95% CI, 3.73, 6.84) and North America (3.92/100 PY; 95% CI, 1.67, 6.17). Reinfection rates among people with HCV test intervals of fewer than 6 months (7.59/100 PY; 95% CI: 5.15, 10.03) were significantly higher than those with more than 6 months test interval (2.88/100 PY; 95% CI: 2.26, 3.50), with an adjusted RR of 1.86 (95% CI, 1.06, 3.13). The adjusted study factors explained 91.03% the of studies' heterogeneity.

**Conclusion:**

HCV reinfection rate was high in successfully treated MSM who were coinfected with HIV-1. A shorter HCV test interval may help to explore more HCV reinfections. HCV reinfection rate studies from HIV-1 coinfected MSM in underdeveloped countries are urgently needed.

**Meta registration:**

PROSPERO: CRD42021285206, URL: https://www.crd.york.ac.uk/prospero/.

## Background

Hepatitis C virus (HCV) and HIV-1 infections have been a major public concern in the world. Since 2013, hepatitis C virus (HCV) had contributed to half of the viral hepatitis mortality, according to a Global Burden of Disease Study (1). In addition, a million HCV-infected individuals were reported to be coinfected with HIV-1 (2). Patients coinfected with HIV-1 and HCV show accelerated liver disease progression and death (3, 4). Meanwhile, liver-related death is a leading cause of non-AIDS mortality among patients with AIDS (5).

With the introduction of highly effective direct-acting antivirals (DAAs) (6), the World Health Organization (WHO) issued its global strategy on viral hepatitis, including an 80% reduction in new HCV infection and a 65% reduction in HCV mortality by 2030 (7). A concept named “HCV micro-elimination” was followed and proposed that HCV diagnosis and treatment uptakes will be carried out in specific sub-populations, such as prisoners, men who have sex with men (MSM), or people who inject drugs (PWID) in networks living with HIV-1 (8). However, the global-wide HCV elimination might be compromised by a high HCV reinfection incidence after successful treatment.

Many studies evaluated the rate of HCV reinfection among specific sub-populations. For example, Hajarizadeh et al. (9) reported an overall rate of HCV reinfection of 5.9/100 person-years (PY) among people with recent drug use. Another multicenter study by Marco et al. (10) reported an HCV reinfection rate of 2.9/100 PY among prisoners. The HCV reinfection rates among HIV-1-infected MSM were reported to vary from 2.54 to15.2/100 PY. To the best of our knowledge, there has been no published systematic study to assess the overall rate of HCV reinfection among HIV-1-infected MSM individuals.

This systematic study seeks to evaluate the rate of HCV reinfection following successful treatments among HIV-1 infected MSM individuals.

## Methods

This study was conducted and reported according to the criteria of Preferred Reporting Items for Systematic Reviews and Meta-Analyses (PRISMA) guideline (11). The review protocol has been registered (PROSPERO: CRD42021285206).

### Search strategy

Two researchers independently conducted literature searches from online databases (PubMed, EMBASE, Medline, and Web of Science) according to procedures provided in Supplementary Text 1. Additional references were identified by manual searching. No language limitations and published studies were updated to October 2021.

### Eligibility criteria

Included studies needed to investigate the reinfection incidence following HCV treatment within the population of HIV-1 coinfected MSM. We included prospective or retrospective studies if they met all the following criteria: (1) the Study population included defined populations of people as MSM and were coinfected with HIV-1; (2) The adopted methods of assessing HCV reinfection following HCV treatment (interferon-based or DAA therapy) had to be explicitly described, including the estimated date and HCV testing method; and (3) clinical trials suitable for the above criteria were also included in this study.

### Data selection and extraction

Two authors independently evaluated all the retrieved articles by title and abstract. A complete text of potentially eligible studies was downloaded. Data were extracted from the selected studies by two independent researchers. Any disagreements were resolved by a third author. Endnote (X9) was used to manage citations and data extraction. The following data were extracted: First author and year, study era, publication type, study design, setting, number of participations, age (mean/median), HIV-1 coinfected, start point, HCV testing schedule, median follow-up time, HCV medication, Re-infection cases, PY, reinfection rate (per 100 PY), and HCV reinfection diagnosis methods (Supplementary Table 1).

### Risk of bias assessment

The quality of reporting was assessed by a qualitative classification according to the Newcastle-Ottawa quality assessment scale for cohort studies, which included eight items with a total score of nine (Supplementary Table 2). Studies were defined to be low risk if the scores were more than 7, moderate risk between 6 and 7, and high risk if <6.

### Statistical analysis

A pooled rate and the corresponding 95% confidence intervals (95% CI) were calculated. Inter-study heterogeneity was appraised using the Q test and I^2^ index. Significant heterogeneity was defined as I^2^ ≥ 50% and *p* < 0.05. Pooled results and the 95%CIs were calculated with a fixed-effect model when I^2^ < 50%; otherwise, a random-effect model was applied (12). Subgroup and meta-regression analyses based on study-level factors shown in **Tables 2**, **3** were conducted to determine the potential sources of heterogeneity.

In the univariate meta-regression, the covariates included median/mean age, median/mean follow-up, study era, study design, publication type, setting, start point, test interval, HCV medication, and risk by NOAS. A final adjusted model by multi-variable meta-regression was performed, including the factors with *p* < 0.1 in the univariate meta-regression. The potential for reporting/publication bias was further visually explored by Begg (12, 13) and presented as a funnel plot. Sensitivity analyses were conducted by the following: (1) sequentially removing each eligible study to evaluate whether a single study dominated the results of the meta-analyses; (2) eliminating high-risk studies; (3) eliminating the non-journal articles; and (4) eliminating the studies without a clear follow-up period. All the analyses were performed using the R software (4.1.1) and the “meta” package. *P* < 0.05 was defined as statistically significant.

## Results

### Study characteristics

**Figure 1:**
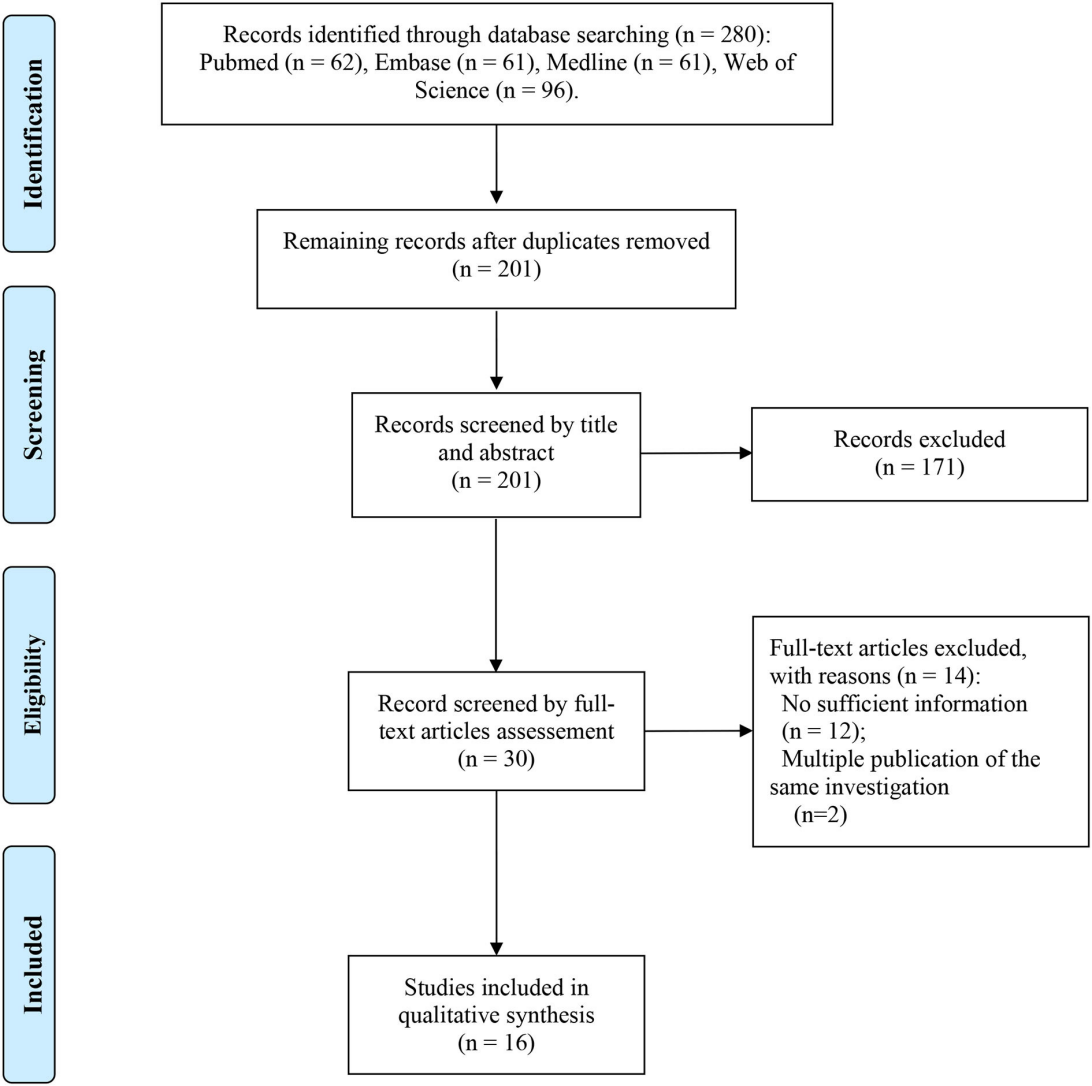
PRISMA flow diagram depicting the literature search and selection strategy. After applying the inclusion and exclusion criteria, a total of 16 articles were included in the final meta-analysis.

Sixteen records with 9,017.2 PY follow-up were eventually included in our quantitative analysis (14–29) (Figure 1, Table 1, Supplementary Table 1). The participants in 11 studies were collected from multi-center while 5 were collected from single-center. Nine studies were designed as prospective and seven were designed as retrospective, and all the studies reported participants with HCV and HIV-1 coinfection. Ten studies were reported from Europe, while 1 study was from Asia. The mean or median age of participants from 34 to 52 years old was reported by 14 studies, and the median follow-up time was from 1.25 to 4.4 years, as reported by 11 studies. Nine out of 16 studies were prospective studies and the other 7 were retrospective studies. HCV treated with DAAs was observed in 5 studies and peg-interferon plus ribavirin therapy in 6 studies. In 4 studies, the HCV reinfection testing started at the end of treatment and 10 studies started at SVR12/24. Diagnosis of HCV reinfection was based on the HCV RNA test (*n* = 14) or HCV RNA-antibody combined test (*n* = 2). Detailed quality assessments were presented in Supplementary Table 1.

**Table 1:** HCV reinfection rate description of the studies included in this meta-analysis.

**First author, Year**	**Study era**	**Cases number**	**Number of participations**	**Reinfection Rate (%)**	**Follow-up, year**	**Person-years**	**Reinfection/100 person-years** **(95% CI)**	**HIV-1 coinfected**
Newsum et al. (29)	Europe	34	122	27.87	1.4	295.5	11.5 (8.2–16.1)	Yes
Boyd et al. (28)	Europe	3	30	10.00	1.25	29.5	0.102	Yes
Huang et al. (27)	Asia	18	128	14.06	4.4	219.5	8.2 (5.2–13.1)	Yes
Chaillon et al. (26)	North America	3	43	6.98	2.7	102.5	2.89 (0.60–8.44)	Yes
Carollo et al. (25)	Europe	12	177	6.78	1.9	202	5.93 (3.37–10.44)	Yes
Pradat et al. (23)	Europe	51	662	7.70	NA	2,007.8	2.54	Yes
Cotte et al. (22)	Europe	15	478	3.14	NA	478	3.1	Yes
Aebi-Popp et al. (21)	Europe	7	43	16.28	NA	201	3.48	Yes
Young et al. (20)	North America	6	84	7.14	1.5	230.8	2.6 (0.6–6.6)	Yes
Pradat et al. (19)	Europe	32	890	3.60	NA	1,250	2.56	Yes
Ingiliz et al. (18)	Oceania	149	606	24.59	3	2,041	7.3 (6.2–8.6)	Yes
Thomas et al. (17)	Europe	19	85	22.35	2.87	320.4	5.93 (3.34–8.26)	Yes
Martin et al. (15)	Europe	32	145	22.07	2.1	400	8.0 (5.7–11.3)	Yes
Lambers et al. (14)	Europe	11	51	21.57	1.3	72.2	15.2 (8–26.5)	Yes
Berenguer et al. (24)	North America	46	278	16.55	1.9	808	5.7 (4.2–7.3)	Yes
EI-Hayek et al. (16)	Oceania	4	63	6.35	NA	84	4.76 (1.74–12.33)	Yes

NA, None data in the included studies; DAAs, Direct-acting antivirals; SVR12/24, sustained virologic response at 12 or 24.

### Pooled HCV reinfection rate in HIV-1 coinfected MSM population

**Figure 2:**
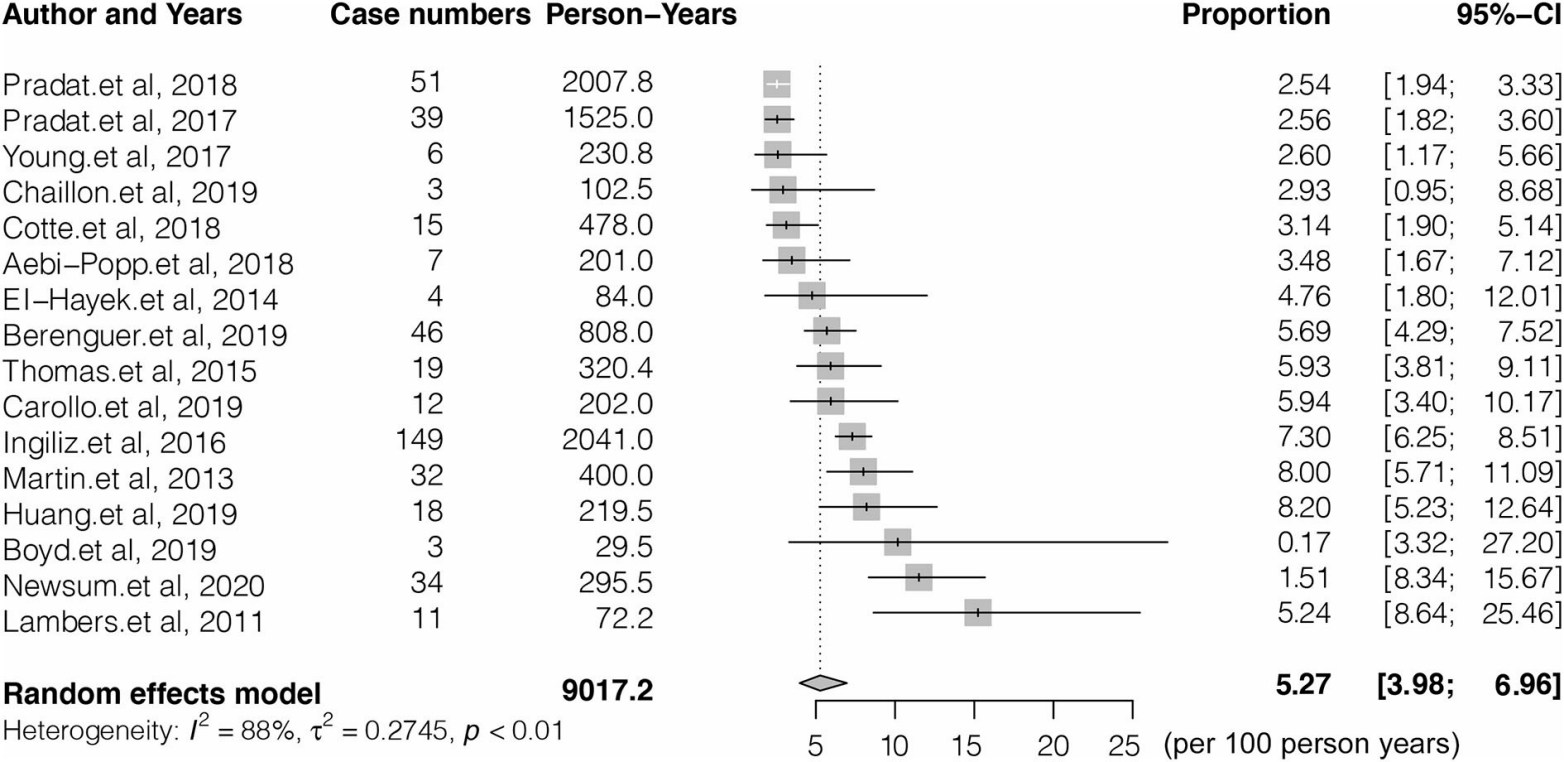
Forest plots of studies for evaluating the HCV reinfection rate in HIV-1 coinfected men who have sex with men (MSM).

Sixteen studies with a total of 9,017.2 PY follow-ups were included to analyze the reinfection rate. High heterogeneity was observed in the analyzed studies (I^2^ = 86.4%, *p* < 0.001) (Figure 2). The pooled reinfection rate was 5.27/100 PY (95% CI, 3.98, 6.96) by random-effects meta-analysis (Figure 2). No publication bias was found based on the funnel plot (Supplementary Figure 2) or Egger's regression (*P* = 0.717, Supplementary Figure 3). Sensitivity analysis by removing a single study showed that the pooled reinfection rate varied from 4.59/100 PY to 5.62/100 PY, no single study had an excessive influence on the pooled prevalence (Supplementary Figure 1). According to the NOS assessments, 6 studies were evaluated as high risk and were excluded from the analysis while the pooled reinfection rate was calculated as 5.21/100 PY (95% CI, 3.59, 7.51) (14, 15, 17, 21, 23, 25) (Supplementary Table 3). As introduced, 6 studies were not peer-reviewed (14, 16, 21, 22, 24, 25) and, hence, were removed before the analysis of the pooled reinfection rate as 5.11/100 PY (95% CI: 3.49, 7.42) (Supplementary Table 3). We also tried to remove studies without a clear follow-up time (19, 21–23), and the pooled reinfection rate was slightly increased to 6.79/100 PY (95% CI: 5.39, 8.53) (Supplementary Table 3).

Sub-group analysis shown in Table 2 indicated the reinfection rate was significantly higher when the HCV reinfection test interval was <6 months (7.59/100 PY; 95% CI: 5.15, 10.03) compared with more than 6 months (2.88/100 PY; 95% CI: 2.26, 3.50). The highest reinfection rate was observed in peg-interferon and (or) ribavirin treatments of the HCV treatment group (7.76/100 PY; 95% CI: 5.77, 10.37) (Table 2).

**Table 2:** Subgroup analysis of HCV reinfection in HIV-1-infected Men who have sex with men (MSM).

**Subgroup**	**Studies numbers**	**Rate (95% CI), per 100 PY**	**I** ^2^	**Model**	***p*** **-value of heterogeneity**	***p*** **-value between subgroups**
**Study era**						
Europe	10	5.28 (3.73, 6.84)	84%	Random	<0.01	0.08
North America	3	3.92 (1.67, 6.17)	68%	Random	0.04	
Oceania	2	7.03 (5.49, 8.57)	11%	Fixed	0.29	
Asia	1	8.20 (4.57, 11.83)	NA	NA	NA	
**Study design**						
Prospective	9	5.92 (3.80, 8.04)	90%	Random	<0.01	0.45
Retrospective	7	4.94 (3.08, 6.80)	79%	Random	<0.01	
**Publication type**						
Journal article	10	5.62 (3.61, 7.63)	91%	Random	<0.01	0.53
Others	6	4.81 (3.30, 6.33)	61%	Random	0.02	
**Setting**						
Multi-center	11	5.08 (3.43, 6.73)	90%	Random	<0.01	0.59
Single-center	5	5.96 (4.81, 7.11)	0%	Fixed	0.77	
**Start point**						
End of treatment	4	8.73 (4.21, 13.26)	83%	Random	<0.01	0.05
SVR12/24	10	4.55 (2.99, 5.91)	87%	Random	<0.01	
**Test interval**						
<6 months	8	7.59 (5.15, 10.03)	92%	Random	<0.01	<0.01
≥ 6 months	7	2.88 (2.26, 3.50)	0	fixed	0.53	
**HCV medication**						
DAAs	5	3.32 (2.24, 4.88)	66%	Random	0.02	<0.01
Peg-interferon and ribavirin	6	7.76 (5.77, 10.37)	72%	Random	<0.01	
Interferon + DAAs	3	3.98 (2.05, 7.56)	89%	Random	<0.01	
**Risk level**						
High risk	7	5.44 (3.41, 7.47)	81%	Random	<0.01	0.05
Moderate risk	6	4.50 (2.04, 6.96)	84%	Random	<0.01	
Low risk	3	7.41 (6.33, 8.48)	0	Fixed	0.79	

PY, person-years; DAAs, direct-acting antivirals; SVR, sustained virologic response; NA, no analysis.

According to the multi-variables meta-regression, the HCV reinfection rate was comparable in the different HCV test interval populations. Compared to people with tested interval of more than 6 months (16, 19–22, 25, 26), those tested interval of <6 months had 1.86 times higher risk of reinfection (aRR: 1.86, 95% CI: 1.06, 3.13; P=0.02) (15, 17, 18, 23, 27–29). The residual I-square of the multi-variables meta-regression was 8.97%, which indicated that the study factors (start point, test interval, and HCV medication) can explain a major proportion of heterogeneity across the included studies (Table 3).

**Table 3:** Meta-regression analysis of study-level factors associated with HCV reinfection rate.

**Group**	**Study no**.	**Unadjusted modle**		**Adjusted modle** *	
		**OR (95% CI)**	**P**	**OR (95% CI)**	**P**
**Median/mean age, per year increase**	14	0.96 (0.90, 1.01)	0.105		
**Median/mean follow-up, per year increase**	11	0.94 (0.62, 2.47)	0.692		
**Study era**					
Asia	1	1			
Europe	10	0.65 (0.21, 2.02)	0.45		
North America	3	0.44 (0.21, 1.61)	0.21		
Oceania	2	0.76 (0.20, 2.90)	0.68		
**Start point**					
End of treatment	4	1		1	
SVR12/24	10	0.48 (0.26, 0.89)	0.02	0.67 (0.36, 1.25)	0.21
**Study design**					
prospective	9	1			
retrospective	7	0.82 (0.45, 1.48)	0.51		
**Setting**					
Multi-center	11	1			
Single-center	3	1.21 (0.65, 2.27)	0.51		
**Test interval**					
≥ 6 months	7	1			
< 6 months	7	2.27 (1.33, 3.85)	0.002	1.86 (1.06, 3.13)	0.02
**Publication type**					
Journal Article	10	1			
others	6	1.23 (0.62, 2.46)	0.54		
**HCV medication**					
DAAs	5	1			
peg-interferon and ribavirin	6	2.32 (1.32, 4.09)	0.003	1.42 (0.69, 2.94)	0.33
Interferon + DAAs	2	1.15 (0.57, 2.31)	0.69	1.22 (0.65, 2.26)	0.55
**Risk by NOAS**					
High risk	7	1			
Moderate risk	6	1.49 (0.69, 3.23)	0.31		
Low risk	3	0.76 (0.41, 1.39)	0.37		

DAAs, direct-acting antivirals; SVR, sustained virologic response; Mixed-Effects Model was utilized to finish the meta-regression analysis;

* adjusted multi-variable meta-regression included variables with P < 0.1 in unadjusted meta-regression. Residual I-square = 8.97%.

### Secondary reinfection rate of HCV in HIV-1-Infected MSM population

Three studies reported a high rate of multiple HCV reinfections. Generally, Martin et al. (15) first observed a higher second HCV reinfection with a rate of 23.2/100 PY vs. 8/100 PY of first HCV reinfection. Inglis et al. (18) presented the first HCV reinfection rate of 7.3/100 PY while the second reinfection increased to 18.8/100 PY. In line with these two studies, Newsum et al. (29) reported that the second, third, or fourth HCV reinfection was 38.3/100 PY, which was even higher than the first reinfection of 9.1/100 PY. Since the original articles did not provide the detailed case number and follow-up dates of the second HCV reinfection, subsequently pooled analysis could not be performed.

### Risk factors for HCV reinfection in HIV-1-infected MSM population

Three of the 17 studies reported associations between potential risk exposures and HCV reinfection in HIV-1-coinfected MSM, including sociodemographic characteristics, HIV-1 clinical characteristics, sexual behavior, drug use, and sexually transmitted infections. According to Huang et al. (27), sexually transmitted infections, such as syphilis, had 10.3 times higher reinfection risk compared to those without syphilis. In addition, Newsum et al. (29), reported that HCV reinfection was associated with nadir CD4 cell count of < 200 cells/mm3 (aHR = 2.22), recent CD4 cell count of < 500 cells/mm3 (aHR = 3.60), receptive condomless anal intercourse (aHR = 4.27), sharing of sex toys (aHR = 4.91), group sex (aHR = 2.80), anal rinsing before sex (aHR = 2.47), and ≥10 casual sex partners in the past 6 months (aHR = 2.81). In addition, Ingiliz et al. (18) found a higher reinfection rate among patients who achieved SVR post-treatment compared to individuals who were spontaneously cleared. HCV infection with an unadjusted HR was 0.62.

## Discussions

We retrieved 16 studies with 3,885 HIV-1-infected MSM and 9,017.2 PY followed-up reported HCV reinfection rates after successful HCV treatment. The HCV reinfection rate significantly varied from 2.54 to 15.2 per 100 PY between the included studies. A random effect model was performed and revealed the pooled HCV reinfection rate (5.27/100 PY; 95% CI: 3.98, 6.96), which was nearly 6 times higher than the HCV incidence among HIV-1-infected MSM (8.46/1000 PY for HIV-1 positive MSM) (30), but slightly lower than PIDU (5.9/100 PY) (9). People who currently use intravenous drugs were more likely to ignore the risk of HCV transmission by intravenous injection, thus, increasing the reinfection rate (31).

HCV reinfection was influenced by many factors. In our subgroup analysis, we found that MSM with DAAs treatment had a high reinfection rate of 3.32/100 PY. However, a higher rate of MSM treated by peg-interferon and ribavirin had a higher reinfection rate of 7.76/100 PY. This may be explained by peg-interferon and ribavirin combined therapy that had a high risk of HCV elimination failure (32). HCV test intervals were highly heterogeneous among the included studies, which may influence the HCV reinfection rate estimation. HCV reinfection rate was significantly higher in the <6 months group (7.59/100 PY) than in the more than 6 years group (2.88/100 PY). After adjustment of the study variables, the HCV reinfection rate was 2.27 times higher in <6 months than more than 6 months group. This result revealed that a long test interval may underrate the real HCV reinfection rate.

Notably, HCV prevalence was reported higher among the underdeveloped regions worldwide, such as Central Asia (3.8%), East Asia (3.7%), and North Africa/Middle East (3.6%), compared with the developed regions (Tropical Latin America, 1.2%; High-income Asia Pacific, 1.4%) (33). A high HCV burden in the underdeveloped regions may contribute to the higher risk of HCV reinfection. However, we did not find any studies implemented in the underdeveloped regions to estimate the HCV reinfection rate among MSM. The lack of research on HCV reinfection in underdeveloped areas may be a key obstacle to HCV elimination around the world.

Compared with the first HCV reinfection rate, we found 3 studies that reported that HIV-1-infected MSM had an almost 4 times higher second HCV reinfection rate. This phenomenon suggested that reinfection populations may consist of some special risk factors of reinfection. Risk factors for HCV reinfection in HIV-1-infected MSM or IDU populations were rarely reported. According to Geddes et al. (34), the incidence of HCV among young women in the United States is rising and likely attributed to several risk factors, such as receptive syringe sharing. However, it is unclear whether these factors also impact HCV reinfection. Several studies reported specific risk factors for HCV reinfection in a cohort of PWID on OAT, including heroin with cocaine or speed, speed alone, or crack alone (35–37). Three studies from Huang et al. (27) also reported several risk factors associated with HCV reinfection and HIV-1-infected MSM (e.g., sociodemographic characteristics, HIV-1 clinical characteristics, sexual behavior, drug use, and sexually transmitted infections). These factors have been inadequately explored due to fewer studies and a small sample size, suggesting that researchers prioritize certain high-risk behaviors during the study. They also highlight the necessity for social educational and motivational interventions by the health institutions.

This study firstly estimated the HCV reinfection rates among HIV-1-infected MSM by meta-analysis but had some limitations. Firstly, high heterogeneity was found during the estimation of the pooled reinfection rates by the random-effect model. Although we performed sub-group analysis, and multi-variables meta-regression to explore the source of heterogeneity, we found that HCV test interval may be one of the main factors attributed to the high heterogeneity. After adjustment, multi-variable meta-regression results revealed a residual I-square of 8.97%, indicating that the factors included in this model could explain a large proportion of heterogeneity across studies. Secondly, the included studies were also limited by the inclusion of heterogeneous study populations, as most populations did not report current injection drug use and HIV-1 treatment status. Thirdly, high-risk level studies according to the NOSA standard were included. We performed a detailed sensitivity analysis including omitting each study and the results of omitting the high-risk studies and non-journal articles revealed no significant alteration in the pooled reinfection rate. Finally, other limitations, such as the study population selection bias and the absence of lost follow-up rate in the included studies, may underestimate the rate of HCV reinfection.

In conclusion, this study revealed a high HCV reinfection rate among HIV-1-infected MSM and demonstrated that HCV test interval is a key factor that may significantly alter the HCV reinfection rate estimation. These results suggest that institutions and researchers must be seriously concerned about the monitoring of HCV reinfection among HIV-1-infected MSM who have successfully undergone HCV treatment. Studies need to be conducted in the underdevelopment regions and explore the special risk factors of HIV-1-infected MSM's HCV reinfection.

## Data availability statement

The original contributions presented in the study are included in the article/Supplementary material, further inquiries can be directed to the corresponding authors.

## Author contributions

YL and PS designed this study and re-edited the manuscript. ZW and PS collected the studies and completed the data analysis. The other authors contributed to the data extraction. All authors contributed to the article and approved the submitted version.

## Funding

This study was supported by the Department of Science and Technology of Sichuan Province, China (No. 2017JZ0039), the Key Research and Development Plan of Chengdu Science and Technology Bureau (2021-YF05-00498), and the Key Research and Development Project of Sichuan Science and Technology Department (2022YFS0600).

## Conflict of interest

The authors declare that the research was conducted in the absence of any commercial or financial relationships that could be construed as a potential conflict of interest.

## Publisher's note

All claims expressed in this article are solely those of the authors and do not necessarily represent those of their affiliated organizations, or those of the publisher, the editors and the reviewers. Any product that may be evaluated in this article, or claim that may be made by its manufacturer, is not guaranteed or endorsed by the publisher.

## Supplementary material

Supplementary Figure 1 Sensitivity analyses to evaluate the contribution of each study to the pooled estimation by excluding each of the studies one after the others.

Supplementary Figure 2 Funnel plot for the publication bias in the overall analysis.

Supplementary Figure 3 Egger's regression teste for publication bias of the included studies (*P* = 0.717).
