# Supplementary figures and images for: Reinfection rate of hepatitis C in HIV-1 positive men who have sex with men: A systematic review and meta-analysis
Source: Front Public Health. 2022 Jul 29;10:855989. doi: 10.3389/fpubh.2022.855989 (PMC9372531; doi:10.3389/fpubh.2022.855989)

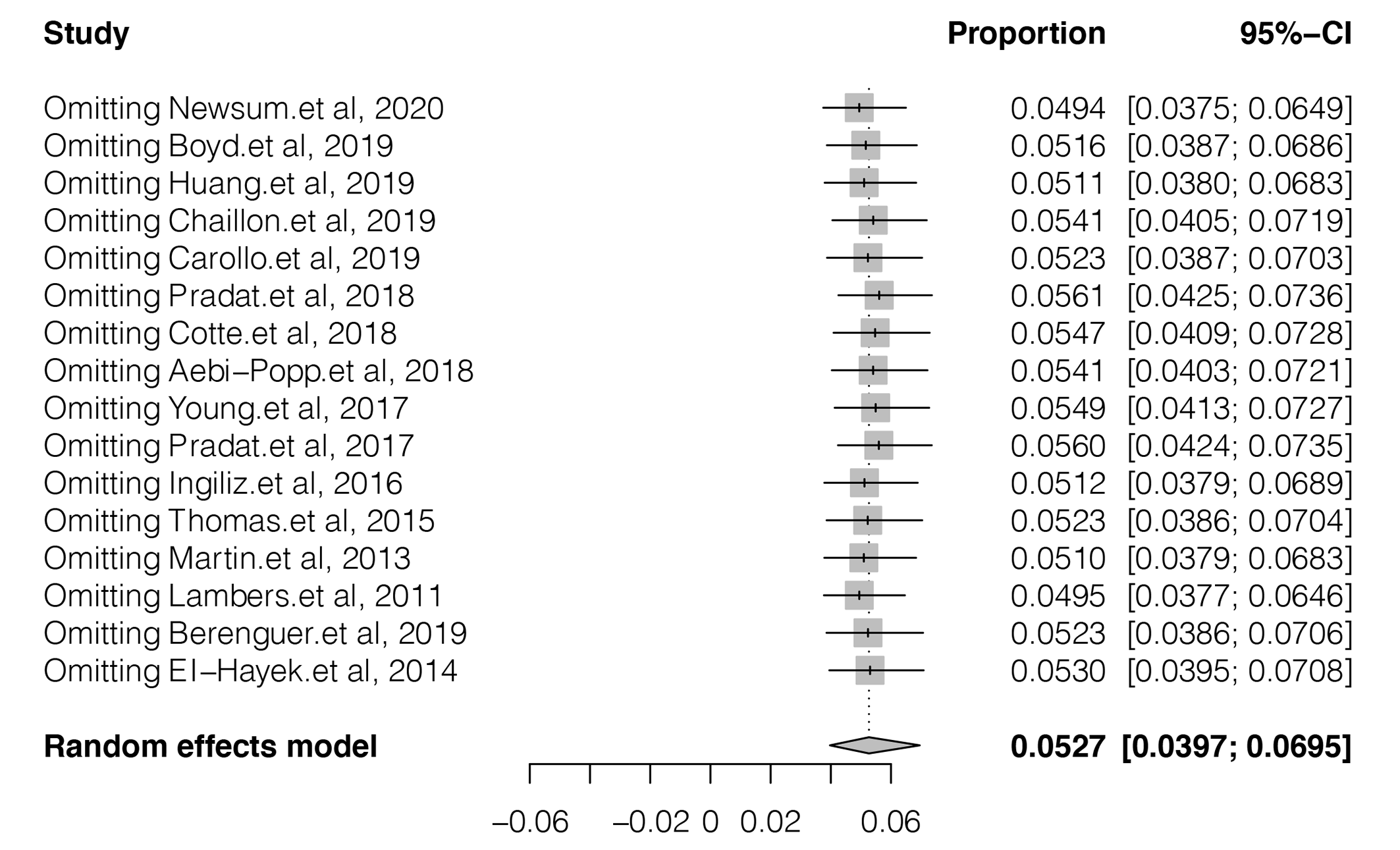

Supplement: Supplementary Figure 1 — Sensitivity analyses to evaluate the contribution of each study to the pooled estimation by excluding each of the studies one after the others. [file Image_1.TIF]

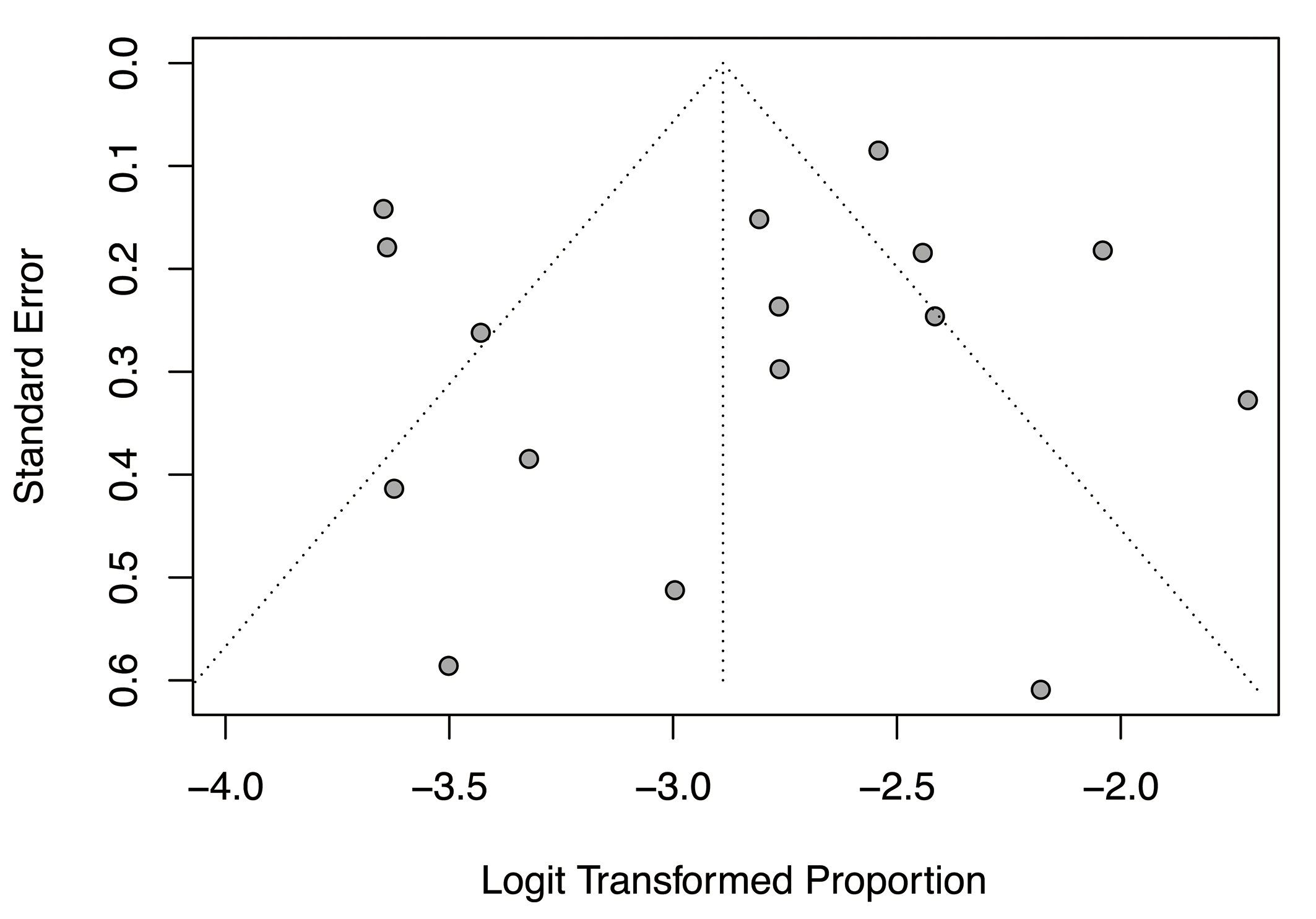

Supplement: Supplementary Figure 2 — Funnel plot for the publication bias in the overall analysis. [file Image_2.TIF]

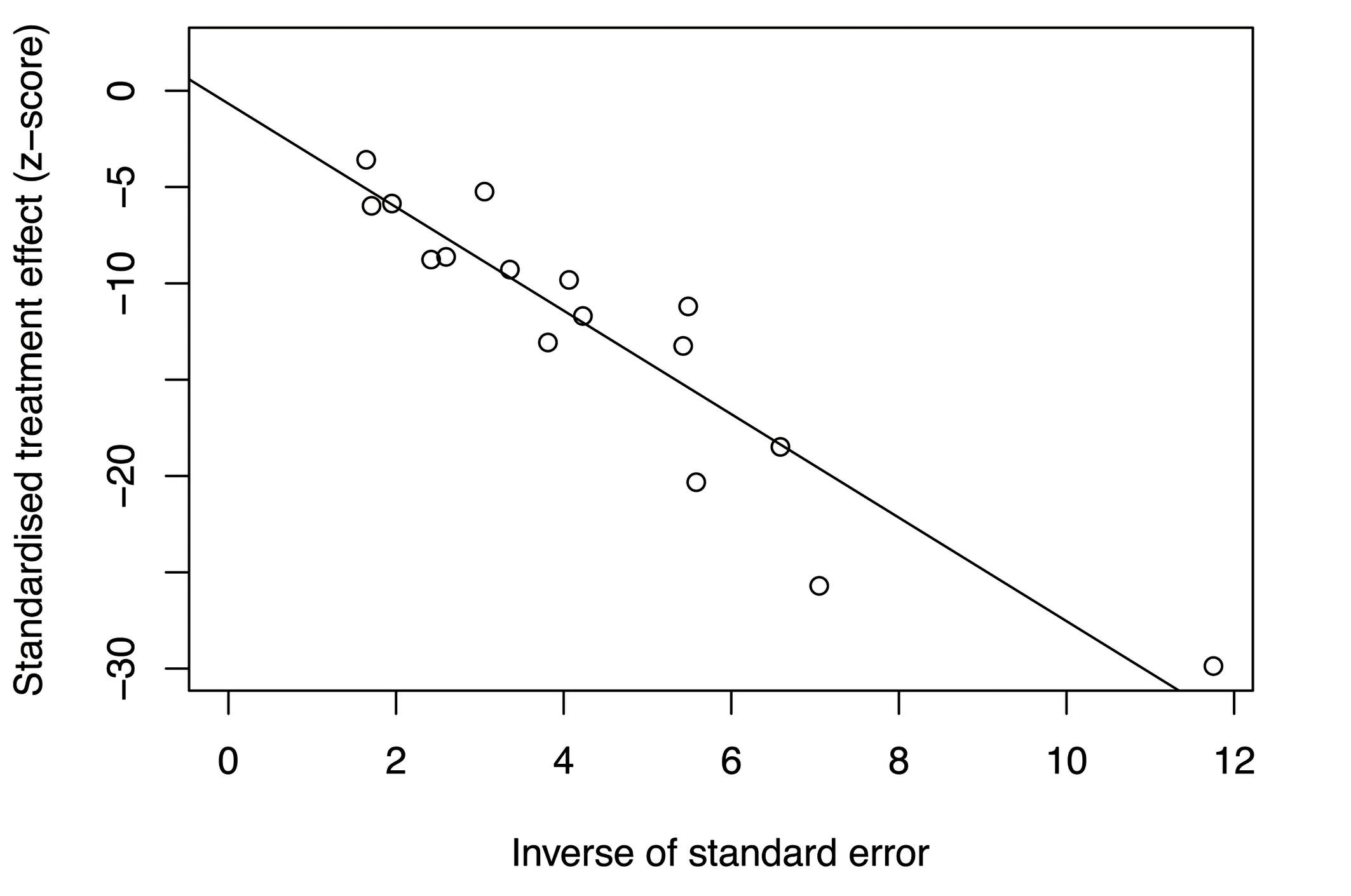

Supplement: Supplementary Figure 3 — Egger's regression teste for publication bias of the included studies (P = 0.717). [file Image_3.TIF]
