# Supplementary material for: Reinfection rate of hepatitis C in HIV-1 positive men who have sex with men: A systematic review and meta-analysis
Source: Front Public Health. 2022 Jul 29;10:855989. doi: 10.3389/fpubh.2022.855989 (PMC9372531; doi:10.3389/fpubh.2022.855989)
Supplement: Supplementary file 4 [file Data_Sheet_1.DOCX]

**Supplemental materials**

**Supplement text 1: Search criteria.**

**Pubmed**

(((MSM[Title/Abstract]) OR (Men Who Have Sex With Men[Title/Abstract])) AND (((HCV[Title/Abstract]) OR (Hepaciviruses[Title/Abstract])) OR (Hepatitis C virus[Title/Abstract]))) AND (reinfection[Title/Abstract])

**Embase**

**1.** MSM. ab. ti.

**2.** Men Who Have Sex with Men. ab. ti.

**3.** 1or 2

**4.** HCV. ab. ti.

**5.** Hepaciviruses. ab. ti.

**6.** Hepatitis C virus. ab. ti.

**7.** 4 or 5 or 6

**8.** reinfection. ab. ti.

**9.** 3 and 7 and 8

**MedLine**

**#1.** TS= ((Men Who Have Sex With Men) OR MSM)

**#2.** TS= (HCV OR Hepaciviruses OR (Hepatitis C virus) )

**#3.** TS= reinfection

**#4.** #3 AND #2 AND #1

**Web of Science**

**#1.** TS= (Men Who Have Sex With Men OR MSM)

**#2.** TS= (HCV OR Hepaciviruses OR Hepatitis C virus)

**#3.** TS= (reinfection)

**#4.** TS= (#1) AND (#2) AND (#3) AND (#4)

Supplemental Table 1. Risk bias evaluation of included studies according to Newcastle-Ottawa quality assessment scale (NOQA).

| Author and Year | Selection | | | | Comparability | Outcome | | | Risk leval  (Score)# |
| --- | --- | --- | --- | --- | --- | --- | --- | --- | --- |
|  | Represen-tativeness of the exposed cohort | Selection of the non-exposed cohort | Ascertain-ment of exposure | Demonstration that outcome of interest was not present at start of study | Comparability of cohorts on the basis of the design or analysis | Assessment of outcome | Was follow-up long enough for outcomes to occur | Adequacy of follow up of cohorts |  |
| Newsum.et al.2020 |  |  |  |  |  |  |  |  | Moderate (7) |
| Boyd.et al.2019 |  |  |  |  |  |  |  |  | Low (8) |
| Huang.et al.2019 |  |  |  |  |  |  |  |  | Low (8) |
| Chaillon.et al.2019 |  |  |  |  |  |  |  |  | Moderate (7) |
| Carollo.et al.2019* |  |  |  |  |  |  |  |  | High |
| Pradat.et al.2018 |  |  |  |  |  |  |  |  | High (4) |
| Cotte.et al.2018 |  |  |  |  |  |  |  |  | Moderate (6) |
| Aebi-Popp.et al.2018 |  |  |  |  |  |  |  |  | High (4) |
| Young.et al.2017 |  |  |  |  |  |  |  |  | Moderate (7) |
| Pradat.et al.2017 |  |  |  |  |  |  |  |  | Moderate (6) |
| Ingiliz.et al.2016 |  |  |  |  |  |  |  |  | Low (8) |
| Thomas.et al.2015 |  |  |  |  |  |  |  |  | High (4) |
| Martin.et al.2013 |  |  |  |  |  |  |  |  | High (4) |
| Lambers.et al.2011 |  |  |  |  |  |  |  |  | High (5) |
| Berenguer.et al.2019 |  |  |  |  |  |  |  |  | Moderate (7) |
| EI-Hayek.et al.2014* |  |  |  |  |  |  |  |  | High |

* Report from Conference; # Studies with scores of <6, 6–7, and >7 were considered as having high, moderate, and low risk of bias, respectively.

Supplemental Table 2. Character description of the included studies.

| **First author, year** | **Publication type** | **Design** | **Setting** | **Start point** | **Age, year, (mean/media)** | **Testing schedule** | **Medication** | **HCV diagnose methods** |
| --- | --- | --- | --- | --- | --- | --- | --- | --- |
| Newsum.et al, 2020 | Journal Article | prospective | multicenter | post-treatment | 46 (40-50) | every six months | peg-IFN and ribavirin: 69.7%; DAA: 22.1%; Spontaneous: 8.2% | HCV antibody and HCV RNA |
| Boyd.et al, 2019 | Journal Article | prospective | multicenter | PTW 12 | 44 (34-52) | every one month | grazoprevir，elbasvir | HCV RNA testing |
| Huang.et al, 2019 | Journal Article | retrospective | Signal-center | SVR12/24 | 34 (7) | 3-6 months | Interferon + DAAs | HCV antibody and HCV RNA |
| Chaillon.et al, 2019 | Journal Article | retrospective | multicenter | SVR12/24 | 50 (42-54) | 1.18 year | Interferon + DAAs | HCV antibody and HCV RNA |
| Carollo.et al, 2019 | Journal Article | prospective | Signal-center | SVR 12 | NA | 6-12 months | DAAs | HCV RNA |
| Pradat.et al, 2018 | Journal Article | prospective | multicenter | SVR 12 | 49 (45-52) | 3-6 months | DAAs | HCV RNA testing |
| Cotte.et al, 2018 | Journal Article | prospective | multicenter | SVR 12 | 41 (50-57) | once a year | DAAs | HCV antibody and HCV RNA |
| Aebi-Popp.et al, 2018 | Short Communication | retrospective | multicenter | post-treatment | 34 (29-45) | once a year | peg-interferon and ribavirin | HCV RNA testing |
| Young.et al, 2017 | Journal Article | prospective | multicenter | SVR 12 | 49 (43-53) | every 6 months | DAAs | HCV RNA testing |
| Pradat.et al, 2017 | Journal Article | retrospective | multicenter | SVR 12 | 52 (46-56) | over 1 year | Interferon + DAAs | HCV RNA testing |
| Ingiliz.et al, 2016 | Journal Article | prospective | multicenter | SVR 12 | 39 (34-44) | every three months | peg-interferon and ribavirin | HCV RNA testing |
| Thomas.et al, 2015 | Journal Article | prospective | Signal-center | SVR 24 | 42 (36-47) | 2.2 months | peg-interferon and ribavirin | HCV RNA testing |
| Martin.et al, 2013 | Journal Article | retrospective | multicenter | post-treatment | 41 | 3-6 months | peg-interferon and ribavirin | HCV RNA testing |
| Lambers.et al, 2011 | FAST TRACK | prospective | multicenter | post-treatment | 44 (9-49) | 3 months | peg-interferon and ribavirin | HCV RNA testing |
| Berenguer.et al, 2019 | Conference Abstract | retrospective | Signal-center | NA | 45 | NA | NA | NA |
| EI-Hayek.et al, 2014 | Conference Abstract | retrospective | Signal-center | NA | NA | 9-12 month | NA | NA |

Supplemental Table 3. Sensitivity analysis of study-level factors associated with HCV reinfection rate.

| Group | No. of studies | Pooled Rate  (95% CI) | Model | I^2^(%) | P Value |
| --- | --- | --- | --- | --- | --- |
| Omitting Non-Journal Articles | 10 | 0.0521(0.0359, 0.0751) | Random | 91.2 | <0.001 |
| Omitting High risk Articles | 9 | 0.0511(0.0349,0.0742) | Random | 88.1 | <0.001 |
| Omitting studies wiout clear follow-up time | 12 | 0.0679(0.0539,0.0853) | Random | 62.9 | 0.002 |
